# The role of satellite DNA-enriched heterochromatic variants in reproductive disorders: Insights from standardized cytogenetic analysis

**DOI:** 10.1007/s10577-026-09800-x

**Published:** 2026-05-08

**Authors:** Sílvia Pires, Isaltina França, Pedro Oliveira, Paula Jorge, Thomas Liehr, Natália Oliva-Teles

**Affiliations:** 1 Centro de Genética Médica Jacinto de Magalhães (CGM), ULS de Santo António, E.P.E., Praça Pedro Nunes, 4099-028 Porto, Portugal; 2 Unit for Multidisciplinary Research in Biomedicine (UMIB), School of Medicine and Biomedical Sciences (ICBAS), University of Porto, Rua Jorge Viterbo Ferreira 228, 4050-313 Porto, Portugal; 3 ITR - Laboratory for Integrative and Translational Research in Population Health, Rua das Taipas 135, 4050-600 Porto, Portugal; 4 EPIUnit – School of Medicine and Biomedical Sciences (ICBAS), University of Porto, Rua Jorge Viterbo Ferreira 228, 4050-313 Porto, Portugal; 5 Department of Microscopy, School of Medicine and Biomedical Sciences (ICBAS), University of Porto, Rua Jorge Viterbo Ferreira 228, 4050-313 Porto, Portugal; 6 Institute of Human Genetics, Jena University Hospital, Friedrich Schiller University, Am Klinikum 1, 07747 Jena, Germany; 7 Faculty of Medicine, University of Porto, Alameda Prof. Hernâni Monteiro, 4200-319 Porto, Portugal

**Keywords:** Chromosomal heteromorphisms, Satellite DNA, Heterochromatic variants, Human infertility, Reproductive genetics

## Abstract

Non-coding DNA, long considered “junk”, is now recognized as a central regulator of genome architecture. Highly repetitive satellite DNA sequences shape heterochromatin and are essential for chromosome stability, segregation, and gene regulation. Pericentromeric heterochromatic variants, or chromosomal heteromorphisms (CHs), have emerged as modulators of human fertility, potentially affecting gametogenesis and early embryonic development. Despite their ubiquity, the functional and clinical significance of CHs remains largely enigmatic. Molecular reference genomes fail to fully capture these repetitive domains, and cytogenetic assessment has shown critical inconsistencies due to the lack of standardized evaluation criteria. To address this gap, we proposed a comparison-based scoring system to reliably identify and characterize CHs. By applying this framework to 300 individuals with idiopathic reproductive disorders and 155 fertile controls, we observed a significantly higher CH frequency in the infertile cohort (2.4-fold increase; p < 0.001). Chromosome 9 variants were the most prevalent (5.3%; p = 0.036), with 1qh+ and 16qh+ as the most common type-specific variants. Cases of recurrent pregnancy loss exhibited the highest CH burden. These results support a significant association between CHs and adverse reproductive outcomes, suggesting that heterochromatic variants may act as predisposing factors for infertility. Importantly, the proposed scoring system addresses critical cytogenetic inconsistencies and provides a comprehensive, reproducible framework that will enhance cross-study comparability and enable future investigations into the structural, functional, evolutionary, and clinical relevance of satellite DNA-enriched heterochromatic regions.

**Supplementary Information:**

The online version contains supplementary material available at 10.1007/s10577-026-09800-x.

## Introduction

Infertility is a complex and heterogeneous condition resulting from structural or functional disorders of the reproductive system, hormonal and immunological dysfunctions, infections, environmental and psychological factors, as well as genetic causes (Jankowska et al. 2025). Identifying its underlying etiology is essential for guiding personalized treatment and reproductive planning and for providing patients with psychological insight into their reproductive challenges (Friedrich and Tüttelmann 2024). Genetic factors account for nearly half of all cases, encompassing chromosomal abnormalities and mono- or polygenic variants that disrupt gametogenesis, fertilization, or early embryonic development (Ding and Schimenti 2021). Although progress has been made in defining the genetic basis of fertility disorders, the contribution of non-coding regions to reproductive function remains poorly understood, despite their increasingly recognized role as key regulators of genomic diversity and gene expression (Liao et al. 2023).

Routine cytogenetics detects variations in large heterochromatic arrays, designated as Chromosomal Heteromorphisms (CHs). These involve size and positional differences in constitutive heterochromatin segments located in centromeric and pericentromeric regions (*e.g.*, 1q12, 9q12, 16q11.2), acrocentric short arms, satellites, stalks, and the Y-chromosome long arm (Liehr 2014). Mainly composed of several families of satellite DNAs (SatDNAs), these sequences are built from tandemly repeated monomers that can organize into higher-order repeat structures, each with distinctive sequence motifs and structural patterns, providing a complex architectural framework within heterochromatic regions (Garrido-Ramos et al. 2025). These repetitive sequences are traditionally considered transcriptionally inactive and phenotypically neutral (Morrison and Thakur 2021). However, emerging evidence demonstrates that SatDNA not only plays a crucial role in the formation and maintenance of heterochromatin, but additionally contributes to proper chromatin organization, centromere function, and epigenetic regulation (Louzada et al. 2020). These sequences can also be transcribed into long noncoding RNAs in germ cells, with variations in these regions potentially affecting chromosome stability, key cellular processes, and reproductive success (Lopez-Pajares 2016; Enriquez and Nechemia-Arbely 2025; Yan and Wang 2025).

Despite advances in long-read sequencing enabling the complete telomere-to-telomere human genome assembly, analysis of SatDNA highly repetitive content remains limited by sparse data and analytical challenges (Warburton and Sebra 2023). Most reference genomes lack full coverage of heterochromatic regions, restricting variant interpretation across individuals and leaving cytogenetic methods as the most effective for large-cohort assessment (Nyaga et al. 2025). However, the current absence of clear guidelines for the cytogenetic evaluation of heteromorphic segments, including the nonexistence of defined size thresholds for CHs and the absence of a universal, objective scoring system, has led to the widespread use of diverse methodologies, resulting in substantial variability in CHs assessment and conflicting findings across published reports (Pires et al. 2024).

We hypothesize that CHs influence human reproductive outcomes and that a standardized analytical framework is essential for robust and clinically relevant research. Accordingly, this study proposes a comprehensive and reproducible comparison-based scoring system for CHs, integrating previous cytogenetic approaches to overcome methodological discrepancies and improve the interpretation of their impact on reproductive health, applied here in a comparative cohort study of infertile individuals and fertile controls.

## Methods

**Fig. 1:**
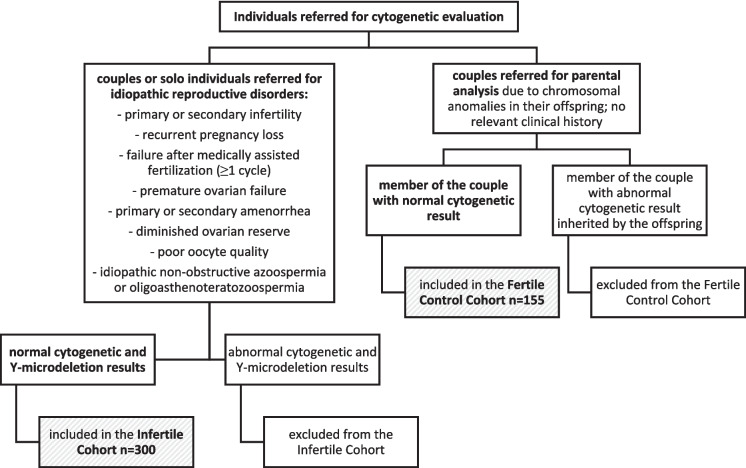
Flowchart outlining the clinical indications and the inclusion and exclusion criteria of participants from both cohorts. Infertile cohort participants presented a mean age of 35.8 years (34.9 ± 5.3 years for females and 36.6 ± 5.6 years for males), and multiple clinical indications per individual were possible

Karyotypes of 300 individuals (145 females and 155 males) referred for cytogenetic evaluation due to idiopathic reproductive disorders and 155 fertile controls (74 females and 81 males) were reanalysed at the Cytogenetics Laboratory, Unidade Local de Saúde de Santo António E.P.E. (ULSSA), Portugal. These samples represent a convenience cohort derived from individuals undergoing routine clinical testing. A flowchart detailing the clinical indications and the inclusion and exclusion criteria for both cohorts is presented in Fig. 1.

### Cytogenetic analysis

Cytogenetic analysis of stimulated leukocytes from peripheral blood samples was performed according to standard protocols. For each sample, a minimum of five metaphase spreads with high-resolution GTL-banded (G-bands using Trypsin and Leishman stain) chromosomes were re-examined using Cytovision 7.4.0.0 (Leica Biosystems®, Nussloch, Germany) software. CHs were evaluated independently by two cytogeneticists and classified according to international cytogenomic nomenclature (ISCN) (Hastings et al. 2024).

The evaluation followed a partially blinded design: one cytogeneticist was involved in routine sample selection and was therefore aware of cohort status, whereas a second cytogeneticist independently assessed all metaphases blinded to cohort information. In cases of discordant classifications, additional metaphase spreads were re-examined and a final classification was reached by consensus after joint review. Prior to the study, both evaluators underwent a calibration phase using 30 test cases to standardize the heteromorphism scoring methodology. Inter-observer agreement was assessed between the two cytogeneticists, and intra-observer agreement was evaluated through repeated assessments performed independently on separate days.

### Heteromorphisms: identification and scoring

**Fig. 2:**
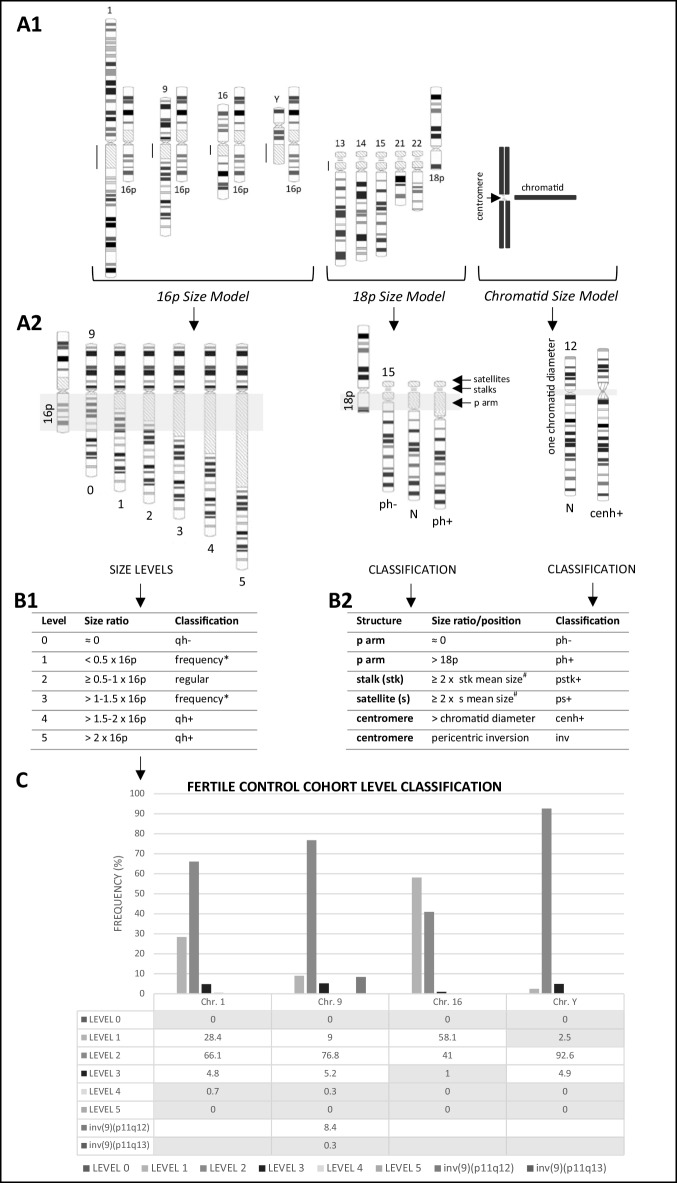
Overview of the scoring framework and classification criteria. **A1—***Size Comparison models* using internal chromosomal references adapted from Liehr (2016): heteromorphic regions of chromosomes 1, 9, 16, and Y are evaluated relative to 16p, all acrocentric short arms relative to 18p, and all centromeric regions relative to the chromatid diameter (chromosomes 16 and 18 ideograms are shown inverted as references). **A2—**Schematic representation using chromosome ideograms, with reference-size regions highlighted in grey (chromosomes 16 and 18 ideograms are shown inverted as references). As examples, the *16p Size Comparison Model* is illustrated for chromosome 9, the *18p Size Comparison Model* for chromosome 15, and the *Chromatid Size Comparison Model* for chromosome 12. Classification levels for the *16p model* are adapted from from Karaca et al. (2020). *N* corresponds to the “normal” chromosome for the evaluated heteromorphic region. **B1—***16p Size Model* classification of heterochromatic regions on chromosomes 1, 9, 16, and Y, using ordinal levels (0–5) based on proportional size relative to 16p. *Intermediate levels are further classified as normal or CH (qh+/−) according to chromosome-specific level frequency distributions for each chromosome in a fertile control population (see C). **B2—***18p and Chromatid Size Models* applied to the classification of acrocentric short arms and centromeric regions (ph+/−, cenh+, and inv variants), respectively. # Secondary constriction structures of acrocentric chromosomes (stalks and satellites) were compared and classified as CHs (pstk+, ps+) based on the mean size of equivalent structures on other acrocentrics within the same metaphase spread. **C—**Level classification and frequency analysis of heteromorphic regions on chromosomes 1, 9, 16, and Y in a fertile control population (population-based calibration). Grey cells indicate levels and inversion defined as CHs for each chromosome, based on low-level frequency (< 3.5%) in a fertile control population (chromosome 1: levels ≥ 4 = 1qh+; chromosome 9: level 0 = 9qh-, levels ≥ 4 = 9qh+; chromosome 16: levels ≥ 3 = 16qh+; Y chromosome: level 1 = Yqh-, levels ≥ 4 = Yqh+; inv(9)(p11q13))

To evaluate all major G-banded heteromorphic segments, including (a) all centromeric regions, (b) pericentromeric blocks located on chromosomes 1, 9, and 16 and the distal portion of the long arm of the Y chromosome, and (c) the short arms, satellites, and stalks of acrocentric chromosomes, a combination of previously described size comparison models was applied. Stable, well-characterized, and proportionally appropriate chromosomal regions were used as internal size references within the same metaphase spread (Fig. 2-A1) (Liehr 2016). Fertile population profiling was performed for heterochromatic segments of chromosomes 1, 9, 16, and Y in order to establish cut-off values for subsequent level assessment and classification of heteromorphisms (Fig. 2-C) (Karaca et al. 2020). These chromosomes harbor large heterochromatic blocks, whereas other heteromorphic regions, although highly variable, lack sufficiently prominent heterochromatin to support robust level stratification:

- *16p Size Comparison Model*: 1q12, 9q12, 16q11.2, and Yq12 heterochromatic blocks were compared to the short arm of chromosome 16 (16p) in the same metaphase spread (Fig. 2-A1). Sizes were scored from level 0 to 5 based on their relative length to 16p (Fig. 2-A2/B1). Most frequent levels in the fertile population were set as thresholds (Fig. 2-C); deviations above/below were classified as CHs, qh+ (increase in long arm heterochromatin), or qh− (decrease in long arm heterochromatin), respectively (Fig. 2-B1). To support the definition of these thresholds, population frequency data were considered, based on previous reports indicating that CHs occur in approximately 2–5% of the general population (Tempest and Simpson 2017); the midpoint of this interval (3.5%) was used as a reference to distinguish common from less frequent variants.
- *18p Size Comparison Model*: Short arms of acrocentric chromosomes (13, 14, 15, 21, and 22) were compared with the short arm of chromosome 18 (18p) in the same metaphase (Fig. 2-A1). Short arms exceeding the length of 18p were classified as ph+ (increase in short arm heterochromatin), whereas apparent absence of the short arm was classified as ph− (decrease in short arm heterochromatin) (Fig. 2-A2/B2). Variations in stalk and satellite structures associated with the secondary constriction of acrocentric chromosomes were classified as CHs if at least twice the mean size of equivalent structures on other acrocentrics in the same metaphase, and designated pstk+ (increase in stalk size) and ps+ (increase in satellite size), respectively (Fig. 2-B2).
- *Chromatid Size Comparison Model*: Average diameter of a single chromosome chromatid was used as a reference for the evaluation of centromere size in all chromosomes (cen) (Fig. 2-A1); enlarged centromeres were classified as cenh+ (increase in centromeric heterochromatin), and pericentric inversions as inv (Fig. 2-A2/B2).

### Statistical analysis—SPSS^® ^Statistics v29.0 software (IBM, Armonk, NY, USA)

Categorical variables were presented as frequencies and percentages. Continuous variables were presented as mean and standard deviation (SD). Inter- and intra-observer agreement were expressed as the proportion of concordant evaluations, and the corresponding 95% confidence intervals (95% CI) were calculated using a binomial distribution. Comparisons for the continuous variables were based on Student’s t test. Categorical variables were studied using the Chi-square test and Fisher’s exact test. Pareto curve analysis (80/20 rule) was applied to identify the most representative clinical indications. Crosstabulation was used to assess homologous size asymmetry. Statistical significance was set at p-value < 0.05.

### Ethical considerations

The study was approved by the Board of Administration and the Ethics Committee of ULSSA, Portugal (TA-DT 2024 084 (075 DEFI 176 CE)); informed consent was waived due to its retrospective and anonymized design.

## Results

### High inter- and intra-observer agreement confirms scoring reliability

A total of 455 cases were independently evaluated by two cytogeneticists. Overall inter-observer agreement was high (92.1%; 95% CI 89.6–94.6), with similar concordance rates observed in infertile patients and fertile controls (Table 1). During the calibration phase using 30 test cases, inter-observer agreement was 90.0%, while intra-observer agreement reached 96.7% for cytogeneticist A and 93.3% for cytogeneticist B (Supplementary Table S1).

**Table 1:** Inter-observer agreement for chromosomal heteromorphisms scoring in the study cohort

Cohort	Total cases (n)	Concordant evaluations n (%)	Discordant evaluations n (%)	95% CI
Infertile patients	300	276 (92.0)	24 (8.0)	88.3–94.6
Fertile controls	155	143 (92.3)	12 (7.7)	86.9–96.0
Total	455	419 (92.1)	36 (7.9)	89.6–94.6

Concordance rates are presented with 95% confidence intervals (95% CI)

### Normalization against internal chromosomal controls, population-calibrated thresholds, and ordinal assessment enable objective CH classification

A total of 310 homologs from the fertile cohort (FC) were scored for large heterochromatic blocks in chromosomes 1, 9, and 16 using the *16p Size Comparison Model*, and 81 Y chromosomes were evaluated under the same reference conditions (Fig. 2-A1/A2/B1). Size level distributions in the FC defined population-based reference patterns (Fig. 2-C). Extreme upper and lower levels were infrequent, allowing objective CHs thresholds to be established in accordance with the frequency-based criterion (3.5%) defined in the Methods. Chromosome-specific cut-offs were set as follows: 1qh+ (levels ≥ 4), 9qh− (level 0)/9qh+ (levels ≥ 4), 16qh+ (levels ≥ 3), and Yqh− (level 1)/Yqh+ (levels ≥ 4). Inversions (inv) were classified independently: inv(9)(p11q12) was common and not considered a CH, whereas inv(9)(p11q13) was rare and defined as a CH (Fig. 2-C, grey cells).

These cut-offs were applied to the infertile cohort (IC), enabling an objective, ordinal, population-calibrated classification of heterochromatin variants in chromosomes 1, 9, 16, and Y as CHs (qh−/+) using the *16p Model*, and were also used to classify pericentric inversion variants in chromosome 9 (inv). By integrating the *18p* and *Chromatid Models* to assess size variations in acrocentric short arms and centromeric regions across all chromosomes, respectively, this approach provides more comprehensive coverage of pericentromeric regions, while reference-based sizing ensures consistency and reduces observer bias.

### Extreme heterochromatin sizes are more frequent in chromosomes 1, 9, 16, and Y of infertile individuals

**Fig. 3:**
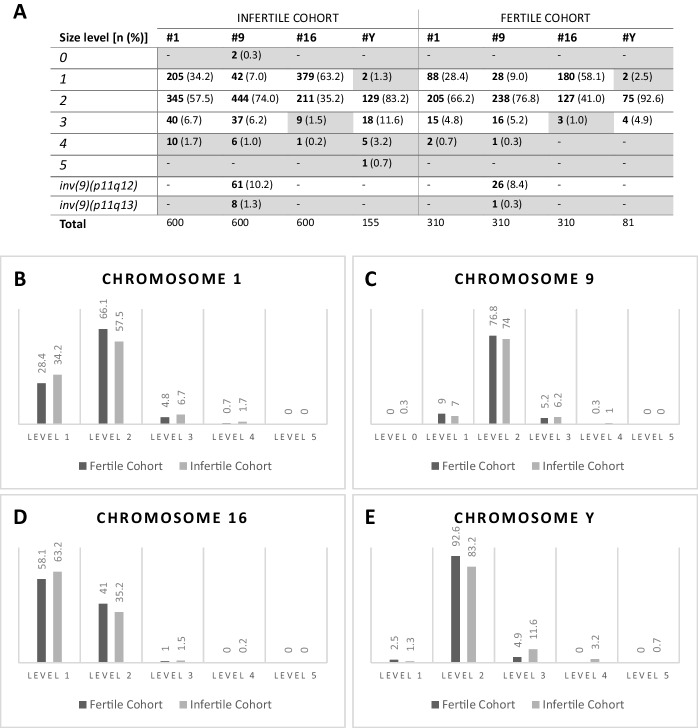
**A**-Level classification and frequency analysis of heteromorphic regions on chromosomes 1, 9, 16, and Y in infertile and fertile cohorts. Grey cells indicate levels and inversion previously defined as chromosomal heteromorphisms (CHs) for each chromosome. **B-E** - Illustration of the frequency of size variants in the heterochromatic regions of chromosomes 1, 9, 16, and Y in fertile and infertile cohorts, assessed using the standardized *16p Size Comparison Model*. Size distribution for each chromosome was independent of cohort, with p-values of 0.061, 0.437, 0.289, and 0.117, respectively

**Fig. 4:**
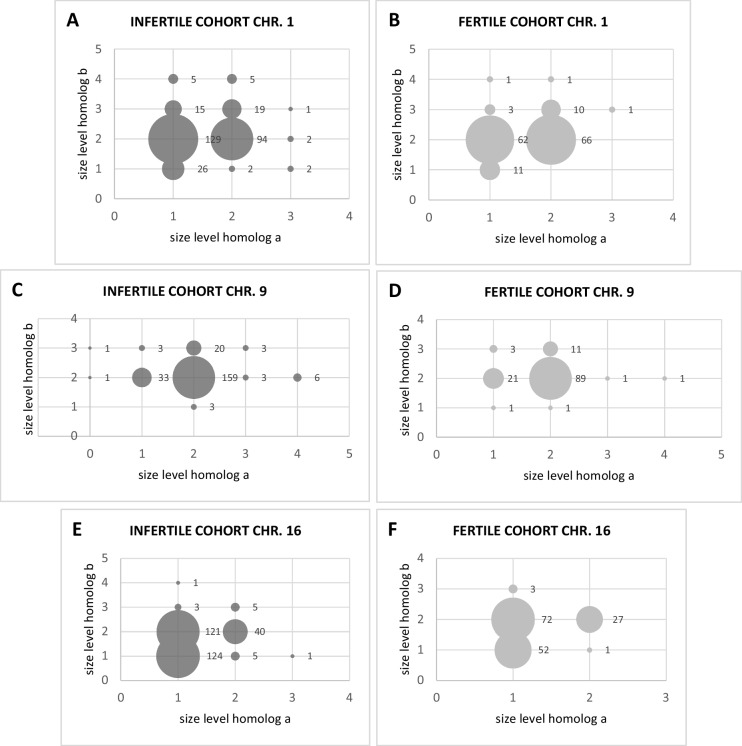
**A-F - **Bubble scatter plots representing cross-tabulated distributions of heterochromatin levels size variants between homologs (a × b) for chromosomes 1, 9, and 16 in fertile and infertile cohorts. Each point corresponds to a specific homolog-level combination (homolog a vs homolog b), with bubble size proportional to its frequency (n). **A-B** For chromosome 1, only the co-occurrence of the most extreme homolog levels (1 and 4) was compared to assess the frequency of size discrepancies between cohorts. **C-D** For chromosome 9, the extreme levels never co-occurred in either the fertile or infertile cohorts. **E-F** For chromosome 16, size variants at levels 3 and 4 (both considered heteromorphisms) were crossed with level 1 variants

Using the population-calibrated thresholds established in 3.2, we evaluated 600 homologs from the IC for chromosomes 1, 9, and 16, as well as 155 Y chromosomes, using the *16p Size Comparison Model*, and compared them with the FC. Figure 3-A summarizes heterochromatin size and positional variations in chromosomes 1, 9, 16, and Y across cohorts, while Fig. 3-B-E illustrates the distribution frequencies of size variants. Larger and smaller size variants and chromosome 9 inversions were more frequent in the IC. Furthermore, crosstabulation of homologous chromosome sizes within individuals revealed that 1q12 size asymmetries were more frequent in the IC (1.7% vs. 0.7%). Conversely, chromosome 16 showed a frequency of 1.7% in the IC, as opposed to 1.9% in the FC. No significant homologous size asymmetry was detected for chromosome 9 in either cohort. Detailed bubble scatter plots representing cross-tabulated distributions of heterochromatin size variants between homologs (a × b) for chromosomes 1, 9, and 16 in both cohorts are provided in Fig. 4.

### Higher CHs frequency in infertile individuals

**Fig. 5:**
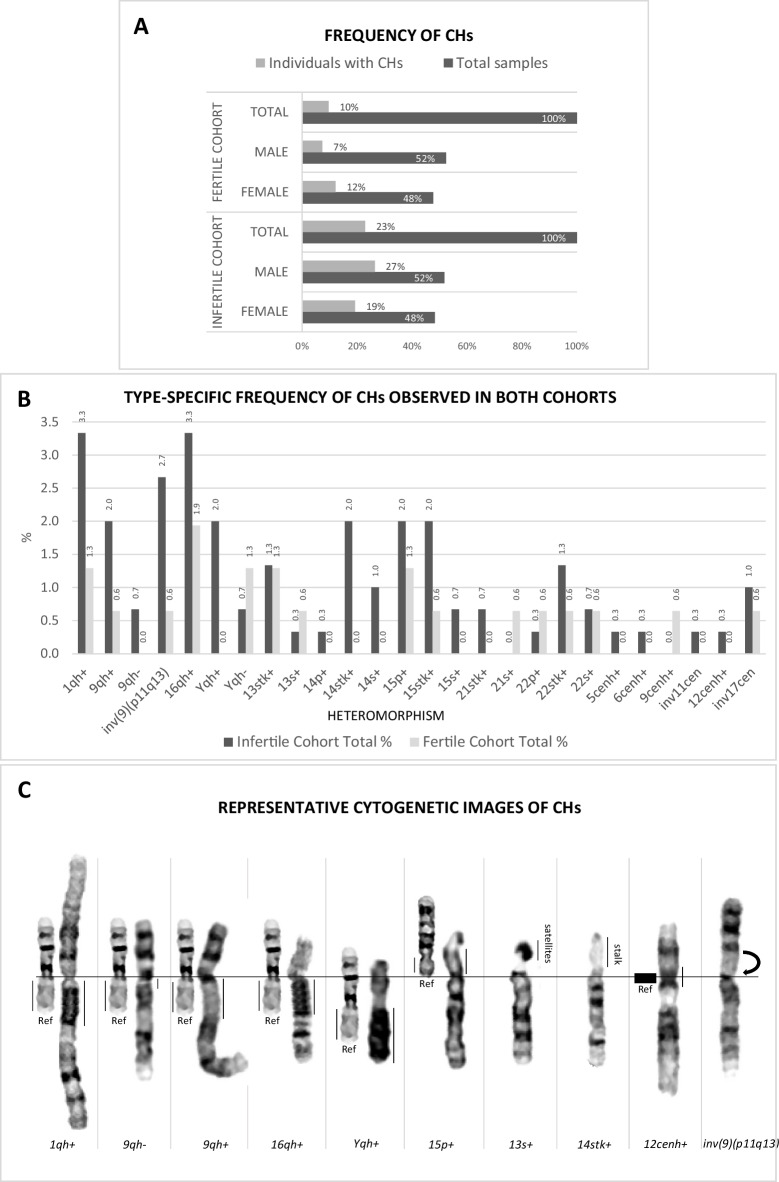
**A - **Frequency of Chromosomal Heteromorphisms detected in fertile and infertile cohorts, expressed as percentages. CHs frequency was 2.4 times higher in the infertile cohort (p < 0.001), with no statistically significant association with sex in either cohort (IC: p = 0.226; FC: p = 0.302). **B **- Graphical representation of the frequency of type-specific CHs across cohorts. **C - **Representative images of chromosomes showing heteromorphisms observed in individuals from the infertile cohort. Cytogenetic nomenclature is indicated below each image. Ref – chromosome 16 ideograms are shown inverted as size references for chromosomes 1, 9, 16, and Y; chromosome 18 ideogram is shown inverted as a size reference for acrocentric short arms; a black block is shown as a reference for chromatid diameter in all centromeric regions. Stalk and satellite structures do not use size reference models; instead, they are compared with the average size of the same structures within the same metaphase spread. The arrow indicates the inversion (inv)

The overall frequency of CHs was 2.4 times higher in the IC (p < 0.001) (Fig. 5-A). Specifically, 23.0% of infertile individuals (28 females, 19.3%; 41 males, 26.5%) carried CHs, versus 9.7% in the FC (9 females, 12.2%; 6 males, 7.4%). In both cohorts, the frequency of CH carriers was independent of sex (IC p = 0.226; FC p = 0.302). In the IC, 89 CHs were identified (in the 69 carriers); 18 individuals (6.0%) had two co-occurring variants, and one (0.3%) had three. In the FC, 21 CHs were detected; two individuals (1.3%) had two co-occurring variants, and two (1.3%) had three (Supplementary Table S2 and Supplementary Figure S1).

### Predominance of chromosome 9 variants in infertile individuals

While 16qh+ (1.9%) was the most frequent in the FC, chromosome 9 variants predominated in the IC (5.3%; p = 0.036) (Fig. 5-B and Table 2). Common IC type-specific variants included 1qh+ and 16qh+ (3.3% each), followed by inv(9)(p11q13) (2.7%). Among infertile females, 16qh+ (4.2%) and 1qh+ (3.5%) predominated; in infertile males, Yqh+ (3.9%) was most frequent, followed by 1qh+ and 9qh+ (3.2% each) (Table 2). Regarding acrocentric chromosome variations exclusively, chromosome 15 variants were common in both cohorts (4.7% infertile, 2.0% fertile). Representative examples of chromosomal heteromorphisms observed in the infertile cohort are shown in Fig. 5-C.

**Table 2:** Type-specific frequency of chromosomal heteromorphisms observed in both cohorts

Heteromorphism	INFERTILE COHORT [n(% of n)]			FERTILE COHORT [n(% of n)]		
	Female n = 145	Male n = 155	Total n = 300	Female n = 74	Male n = 81	Total n = 155
*1qh*+	5 (3.5)	5 (3.2)	10 (3.3)	1 (1.4)	1 (1.2)	2 (1.3)
*9qh*+	1 (0.7)	5 (3.2)	6 (2.0) ^a^	1 (1.4)	-	1 (0.7)
*9qh-*	2 (1.4)	-	2 (0.7) ^a^	-	-	-
*inv(9)(p11q13)*	4 (2.8)	4 (2.6)	8 (2.7) ^a^	-	1 (1.2)	1 (0.7)
*16qh*+	6 (4.2)	4 (2.6)	10 (3.3)	1 (1.4)	2 (2.5)	3 (1.9)
*Yqh*+	n/a	6 (3.9)	6 (2.0)	n/a	-	-
*Yqh-*	n/a	2 (1.3)	2 (0.7)	n/a	2 (2.5)	2 (1.3)
*13stk*+	-	4 (2.6)	4 (1.3)	1 (1.4)	1 (1.2)	2 (1.3)
*13s*+	-	1 (0.7)	1 (0.3)	1 (1.4)	-	1 (0.7)
*14p*+	1 (0.7)	-	1 (0.3) ^b^	-	-	-
*14stk*+	3 (2.1)	3 (1.9)	6 (2.0) ^b^	-	-	-
*14s*+	2 (1.4)	1 (0.7)	3 (1.0) ^b^	-	-	-
*15p*+	2 (1.4)	4 (2.6)	6 (2.0)	1 (1.4)	1 (1.2)	2 (1.3)
*15stk*+	2 (1.4)	4 (2.6)	6 (2.0)	1 (1.4)	-	1 (0.7)
*15s*+	-	2 (1.3)	2 (0.7)	-	-	-
*21stk*+	1 (0.7)	1 (0.7)	2 (0.7)	-	-	-
*21s*+	-	-	-	-	1 (1.2)	1 (0.7)
*22p*+	-	1 (0.7)	1 (0.3)	-	1 (1.2)	1 (0.7)
*22stk*+	4 (2.8)	-	4 (1.3)	1 (1.4)	-	1 (0.7)
*22s*+	1 (0.7)	1 (0.7)	2 (0.7)	1 (1.4)	-	1 (0.7)
*5cenh*+	1 (0.7)	-	1 (0.3)	-	-	-
*6cenh*+	-	1 (0.7)	1 (0.3)	-	-	-
*9cenh*+	-	-	-	-	1 (1.2)	1 (0.7)
*inv11cen*	1 (0.7)	-	1 (0.3)	-	-	-
*12cenh*+	-	1 (0.7)	1 (0.3)	-	-	-
*inv17cen*	-	3 (1.9)	3 (1.0)	-	1 (1.2)	1 (0.7)
Total	36	53	89	9	12	21

The total number of heteromorphisms exceeds the total number of carriers due to the possible coexistence of more than one type of heteromorphism in the same individual; n/a—not applicable; grey lines indicate rare heteromorphic variants, involving enlargement (*cenh*+) and inversions (*inv#cen*) in the centromeric regions; ^a^ P-value < 0.05 for the sum of variants on chromosome 9; ^b^ P-value < 0.05 for the sum of variants on chromosome 14

### CHs co-occurrences frequently involve acrocentric chromosomes

Among the 19 CH co-occurrences observed in the IC, only the 15p+/Yqh+ and 16qh+/22stk+ combinations were detected in more than one individual (two cases each). In 17 cases, at least one acrocentric chromosome was involved, with chromosome 15 appearing most frequently in 8 of the 19 co-occurrence events. In the FC, all CH co-occurrences were unique, although variants involving chromosomes 9 and Y were each observed in two of the four cases. Detailed data are provided in Supplementary Table S2 and Supplementary Figure S1.

### CHs burden differs by clinical indication

**Fig. 6:**
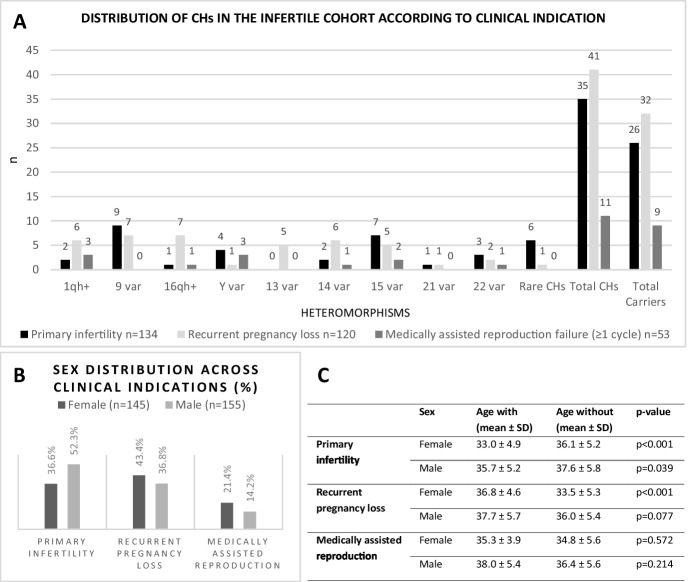
**A - **Distribution of different Chromosomal Heteromorphisms according to clinical indication in the infertile cohort. Only the clinical indications with the highest representation are shown based on Pareto curve analysis. *n* represents the absolute number of cases. The total number of heteromorphisms across clinical indications exceeds the total number of heteromorphisms detected in the study population due to the possible presence of multiple clinical indications in the same individual. **B - **Graphical representation of sex distribution according to clinical indication. **C - **Sex-stratified mean age within the infertile cohort, comparing individuals with and without each clinical indication, including statistical significance; “Age with” refers to individuals presenting the indication and “Age without” to those without it

In the IC, primary infertility (n = 134) and recurrent pregnancy loss (n = 120) were the most represented phenotypes (Fig. 6-A and Supplementary Table S3). Recurrent pregnancy loss showed the highest CH burden (26.7% of carriers), with predominance of 16qh+, 1qh+, and chromosome 9 variants. This phenotype was more prevalent among females (43.4% vs. 36.8%) (Fig. 6-B), with statistical significance found for the mean age of affected females (36.8 ± 4.6 years), which was higher compared to females without recurrent pregnancy loss (p < 0.001) (Fig. 6-C). Primary infertility (with 19.4% of carriers) frequently involved variants on chromosomes 9 and 15 (Fig. 6-A). Sex and the total number of CHs detected in this group were not statistically independent (p = 0.016), with CHs being more frequent in males. The condition was observed in 52.3% of men and 36.6% of women, indicating a statistically significant association with sex (p = 0.006) (Fig. 6-B). Mean age of affected females (33.0 ± 4.9 years) was significantly lower than that of females without primary infertility (p < 0.001), and mean age of affected males (35.7 ± 5.2 years) was significantly lower than that of males without primary infertility (p = 0.039) (Fig. 6-C). Failure in medically assisted reproduction techniques is the third most represented clinical indication (n = 53), with 17.0% of carriers, predominantly showing 1qh+ and Y chromosome variants. No statistically significant differences were observed for CH frequency, age, or sex in this group (Fig. 6-A/B/C).

## Discussion

Advances in molecular sequencing have greatly improved our understanding of the genetic basis of reproductive disorders (Ding and Schimenti 2021). Yet, despite growing recognition of non-coding DNA as a key regulator of genomic diversity and gene expression, its role in reproductive pathophysiology remains largely unexplored (Spielmann and Mundlos 2016; Liao et al. 2023). This gap is particularly pronounced in highly repetitive, SatDNA-enriched regions, where sequence complexity, assembly challenges, and the scarcity of population-scale data hinder discrimination between normal variation and disease-associated alterations (Garrido-Ramos et al. 2025). Although long-read and telomere-to-telomere assemblies improve resolution, accurate quantitative analysis of large satellite arrays remains technically demanding, especially in centromeric regions, due to residual sequencing errors, extreme repeat homogeneity, and substantial structural variability across individuals, limiting routine implementation in laboratory and diagnostic workflows (Altemose et al. 2022; Minton 2024). The intricacy of these segments makes cytogenetic techniques still indispensable for effectively covering centromeric and pericentromeric domains, providing informative direct observations of expansions and contractions in the landscape of repetitive sequences, and enabling the characterization of SatDNA features (Liehr 2021). In this context, cytogenetics-based cohort studies applying objective criteria are essential to generate large-scale, reproducible data to clarify correlations between microscopically observable genetic variation and human infertility (Hanson and Hotaling 2018).

Many cytogenetic reports evaluating CHs rely on heterogeneous methodologies, often restricted to a subset of chromosomes or lacking detailed assessment criteria, which reduces comparability; in our previous comprehensive review (Pires et al. 2024), we found that a substantial proportion of published studies did not clearly specify the scoring method used for heterochromatin assessment. Among those that did, considerable variability was observed even within nominally identical methodological frameworks, including differences in reference regions and measurement criteria. This heterogeneity likely contributes to the conflicting frequency estimates and inconsistent associations reported for CHs and reproductive outcomes (Madan 2025). As highlighted in our critical review, commonly used approaches present intrinsic limitations: the *16p Size Comparison Model* covers only four chromosomes (1, 9, 16, and Y), restricting the evaluation of several heterochromatic regions; the *Twice the Size of the Homologous Method* does not account for variants present simultaneously in both homologs; and the *Linear Measurement Method* is influenced by differences in chromosomal resolution and metaphase quality.

To minimize karyotyping subjectivity and expand coverage, we developed the present scoring framework through critical integration of previously published models rather than adopting a single approach. Specifically, we combined the *16p, 18p*, and *Chromatid Size Comparison Models* proposed by Liehr (2016), using cytogenetically stable internal reference regions to reduce resolution-dependent variability and enabling a broad evaluation of all main heterochromatic regions. Furthermore, since “normal” size ranges for heterochromatic regions are not well defined, the incorporation of size- and positional-level characterization of the major and well-recognized heterochromatin blocks of chromosomes 1, 9, 16, and Y, as described by Karaca et al. (2020), enabled us to profile a fertile population and establish population-specific thresholds for classifying these heterochromatin variants as CHs. Within this approach, scoring consistency was improved through three key methodological elements: (a) normalization against internal chromosomal size controls; (b) standardized ordinal categorization replacing purely observational descriptions; and (c) population-based calibration of scoring thresholds.

Building on this framework, the increased frequency of CHs observed in infertile individuals reinforces prior associations with reproductive failure, with studies reporting a 3 to 5 fold higher incidence of CHs in infertile populations compared to fertile controls (Tempest and Simpson 2017). Although our results indicated a higher incidence of CHs in infertile males than in infertile females, this difference was not statistically significant. In the IC, chromosome 9 variants were the most common, and 1qh+ was the predominant type-specific CH, showing substantial representation in both males and females, consistent with previous links to human infertility (Nakamura et al. 2001; Sipek et al. 2014; Cheng et al. 2017). By contrast, 16qh+ was highly prevalent across cohorts, suggesting a limited association with infertility-related phenotypes. Although chromosome 16 is reported as one of the most aneuploidy prone chromosomes in preimplantation embryos (Carioscia et al., 2026), our data indicate that pericentromeric heterochromatin alone is unlikely to explain these meiotic missegregation events. Other factors, including recombination patterns, spindle assembly checkpoint fidelity, centromere cohesion, and epigenetic modifications, are also likely to contribute to these high aneuploidy rates. The euchromatic content adjacent to the heteromorphic segment may influence phenotypic impact, potentially explaining why certain variants are more strongly associated with fertility impairment. Supporting this thought, Bache et al. (2004) identified several candidate genes involved in spermatogenesis within the 1q21 band, located adjacent to the heterochromatic region. In the same line, Wang et al. (2019) reported TESK1, a testis-specific protein kinase gene expressed in germ cells, located at 9p13.3. By contrast, euchromatic regions adjacent to 16qh do not contain well-characterized fertility-associated genes; some *loci* in this region are linked to neurodevelopmental phenotypes (Kim et al. 2026).

Size analysis of heterochromatic blocks in chromosomes 1, 9, 16, and Y revealed that both enlarged and reduced variants were more frequent in the IC, suggesting the existence of size thresholds defining the clinical relevance of heterochromatin expansion or reduction. Notably, size discrepancies between homologous chromosomes, as observed in chromosome 1 within the IC, could interfere with meiotic pairing or segregation. Altered heterochromatic regions may affect centromere function and homologous recognition, potentially compromising fertility (Anton et al. 2020). In line with this hypothesis, Antonelli et al. (2000) demonstrated that excessive Y-chromosome heterochromatin may impair X-Y pairing and activate meiotic checkpoints, ultimately leading to spermatocyte loss.

Regarding clinical indications, we observed the highest CH burden in the recurrent pregnancy loss group, which was more prevalent among females and associated with a statistically significant higher mean age. This finding reinforces the notion that heterochromatic variants may contribute to reproductive instability, supporting their association with increased risk of unbalanced gamete production and fetal malformations (Cheng et al. 2017; Pang et al. 2024; Sinha et al. 2025). Our results are also consistent with Cheng et al. (2017), who reported a positive association between female age, the presence of CHs, and the incidence of spontaneous abortion. In contrast, primary infertility showed a statistically significant higher CH burden in males, consistent with Sipek et al. (2014), who suggested that heteromorphisms may have sex-specific effects, possibly linked to the Y chromosome’s role in meiotic germ cell division.

While our results, together with previous studies, suggest a potential role for specific size and positional heteromorphic variants in genome regulation during reproduction, evidence remains fragmented and often conflicting (Tempest and Simpson 2017). In a recent review by Madan (2025), surveying published studies on heteromorphisms and their possible clinical impact, 38 studies reported heterochromatic variants as potentially pathogenic, whereas 16 found no evidence of harmful effects. In addition, limited data on the prevalence and type-specific distribution of CHs across populations, compounded by ethnic variability and methodological differences, further challenge the interpretation of these variants (Bhasin 2005; Wyandt et al. 2017). Moreover, the underlying mechanisms by which CHs may affect fertility are still poorly understood. Heteromorphisms are hypothesized to influence reproduction through multiple pathways: (a) by disrupting chromosome pairing and centromere positioning and function, thereby promoting nondisjunction, and impairing gamete formation (Anton et al. 2020); (b) by inhibiting or modulating the expression of key reproductive genes via *Position-effect variegation* (Liang et al. 2023); (c) by altering chromatin dynamics, 3D genome organization, and transitions between heterochromatin and euchromatin states, affecting the accessibility of regulatory elements and transcription factors critical for reproduction (Dey et al. 2022; Liao et al. 2023); (d) by *Satellite association* through the metaphase spatial proximity and the high A-T content of acrocentric chromosomes, which may influence nucleolus organization and chromosomal interactions, contributing to the emergence of chromosomal abnormalities (Antonarakis 2022).

In addition to these mechanisms, recent studies have revealed a direct role for SatDNAs in reproductive processes. Large SatDNA arrays can be transcribed into long noncoding RNAs that are expressed during early embryogenesis and contribute to cellular and tissue differentiation (Lopez-Pajares 2016; Ariffen et al. 2024). Furthermore, these noncoding RNAs have been shown to act as critical regulators of post-transcriptional gene expression during spermatogenesis and oogenesis (Robles et al. 2019), and their widespread presence in germ cells throughout meiosis, spermatogenesis, and early post-fertilization development has led to their proposal as potential fertility biomarkers, particularly in male infertility (Yan and Wang 2025). Supporting this view, studies in mouse embryonic stem cells have shown that dysregulation of satellite repeat transcripts alters heterochromatin condensation and chromosome stability, highlighting a conserved mechanism by which SatDNA perturbation can affect genome integrity and potentially influence reproductive outcomes (Novo et al. 2022). In this context, we speculate that long noncoding RNAs derived from size-expanded SatDNA segments could disturb cellular metabolism, disrupt meiotic progression, and impair fertility, with analogous consequences potentially resulting from reduced heterochromatin levels.

Based on our experience, the reproductive impact of heteromorphisms is unlikely to depend on a single mechanism in all cases. Instead, it may involve one or more pathways, and identifying the predominant mechanism will likely require case-by-case analyses. Additionally, the analysis of co-occurring variants should be considered in light of the *Two-Hit Model*, which proposes that multiple benign variants in an individual may collectively contribute to a clinical phenotype (Liehr 2016). In our results, the overrepresentation of acrocentric chromosomes among co-occurring variants raises the possibility that similarities in repetitive sequence architecture and A–T-rich nucleotide composition may facilitate interchromosomal dynamics with potential cumulative phenotypic effects.

Emerging evidence further indicates that CHs and the content of SatDNA repeats are associated with a range of clinical phenotypes beyond human infertility (Black and Giunta 2018; Ershova et al. 2020; Liehr 2022; Ariffen et al. 2024). For example, Ershova et al. (2020) found that individuals with schizophrenia exhibit significantly reduced satellite III (1q12) and altered ribosomal repeat copy numbers. Ariffen et al. (2024) reported amplification of specific satellite DNA families in prostate cancer, correlating with increased tumor aggressiveness. Black and Giunta (2018) showed that size and structural variations in centromeric and pericentromeric satellites induce chromosomal fragility by challenging DNA replication and repair, contributing to chromosomal instability syndromes. Together, these studies underscore the pathogenic potential of size and copy number variations in SatDNA, supporting multiple underlying mechanisms.

Considering human fertility, although CHs are not the sole contributors to reproductive failure, we propose that they may act as significant predisposing factors. In this regard, accurate identification and reporting of heterochromatic variants could support genetic counselling and, in the future, potentially inform personalized therapeutic approaches (Jankowska et al. 2025). Possible clinical applications, such as embryo selection in couples with recurrent miscarriage or sperm cytogenetic aneuploidy screening in male carriers of CHs, may be considered on an individual basis (Anton et al. 2020). However, the current absence of a universal CHs scoring system and clear reporting guidelines contribute to assessment discrepancies, hindering functional and clinical interpretation. Furthermore, molecular cytogenetic techniques have not been systematically applied to the evaluation of infertile phenotypes. This represents a general limitation in the field and also applies to the present study, meaning that cryptic euchromatic imbalances cannot be excluded. As a result, the ability to draw robust and unbiased conclusions may be compromised. In this context, positional data obtained through fluorescent in situ hybridization are additionally essential for accurately mapping SatDNA families and distinguishing heterochromatic subvariants (Louzada et al. 2020). A further limitation is that analyses were performed on stimulated leukocytes, which are mitotic cells; reproductive disorders may involve meiosis-specific mechanisms not fully captured by mitotic cytogenetic evaluation, and therefore extrapolation to meiotic processes should be made with caution.

While further in-depth molecular studies of SatDNA-rich non-coding regions are essential to fully elucidate their roles in human pathologies, our study reinforces the association between CHs and human infertility and highlights that expert cytogenetic analyses remain an indispensable tool for visualizing chromosome and chromatin dynamics, enabling the characterization of complex repetitive landscapes beyond what sequencing alone can achieve. Although external validation will be necessary to formally quantify the inter-study reproducibility of the proposed CH scoring framework, its structured, reference-based, and population-calibrated design directly addresses key limitations identified in prior studies, providing a coherent and robust strategy for evaluating SatDNA-enriched heterochromatic regions in clinical and evolutionary genomics research. By profiling a fertile cohort, this approach establishes reference values for heteromorphic regions, enabling objective comparisons and the identification of potential links between specific variants and reproductive outcomes in infertile individuals. When applied to large cohorts and integrated with advanced long-read sequencing technologies and specialized bioinformatic tools for satellite repeat content analysis, this framework offers an opportunity to refine variant interpretation, clarify functional significance, and improve diagnostic accuracy, underscoring the continued relevance of cytogenetics in the era of high-throughput genomics.

## Supplementary Information

Below is the link to the electronic supplementary material.

Supplementary file1 (PDF 876 KB)

## Data Availability

Datasets generated and/or analysed during the current study are not publicly available due to privacy and ethical restrictions, but de-identified data may be available from the corresponding author on reasonable request and pending ethical approval.

## Abbreviations

*cen*: Centromere
*cenh+*: Increase in constitutive heterochromatin at the centromere
*CH*: Chromosomal heteromorphism
*CHs*: Chromosomal heteromorphisms
*FC*: Fertile cohort
*GTL*: G-bands using Trypsin and Leishman stain
*IC*: Infertile cohort
*inv*: Pericentric inversion
*ISCN*: International System for Human Cytogenomic Nomenclature
*p*: Short arm of a chromosome
*ph+*: Increase in short arm constitutive heterochromatin
*ph−*: Decrease in short arm constitutive heterochromatin
*ps+*: Increased satellite size
*pstk+*: Increased stalk size
*q*: Long arm of a chromosome
*qh+*: Increase in long arm constitutive heterochromatin
*qh−*: Decrease in long arm constitutive heterochromation
*SatDNA*: Satellite DNA
*SD*: Standard deviation
*SPSS*: Statistical Package for the Social Sciences
*ULSSA*: Unidade Local de Saúde de Santo António
