# Supplementary material for: The role of satellite DNA-enriched heterochromatic variants in reproductive disorders: Insights from standardized cytogenetic analysis
Source: Chromosome Res. 2026 May 8;34(1):9. doi: 10.1007/s10577-026-09800-x (PMC13156139; doi:10.1007/s10577-026-09800-x)
Supplement: Supplementary file 1 — Supplementary file1 (PDF 876 KB) [file 10577_2026_9800_MOESM1_ESM.pdf]

Supplementary Table S1. Reproducibility of heteromorphism scoring during evaluator calibration

Assessment	Cases evaluated (n)	Concordant evaluations n (%)	95% CI
**Inter-observer agreement (Cytogeneticist A vs Cytogeneticist B)**	30	27 (90.0)	73.5–97.9
**Intra-observer agreement (Cytogeneticist A)**	30	29 (96.7)	82.8–99.9
**Intra-observer agreement (Cytogeneticist B)**	30	28 (93.3)	77.9–99.2

Note: Calibration was performed prior to the study to standardize the heteromorphism scoring methodology. Inter- and intra-observer agreement were calculated as the proportion of concordant evaluations, with 95% confidence intervals (95% CI) estimated using a binomial distribution. Intra-observer agreement was assessed by repeated evaluations conducted independently on separate days.

Supplementary Table S2. Co-occurrence of Chromosomal Heteromorphisms (CHs)

	INFERTILE COHORT			*FERTILE COHORT*		
HETEROMORPHISM Co-OCCURRENCE	*FEMALE (n=145)*	*MALE (n=155)*	*TOTAL (n=300)*	*FEMALE (n=74)*	*MALE (n=81)*	*TOTAL (n=155)*
5cenh+/21stk+	1	-	1	-	-	-
6cenh+/12cenh+	-	1	1	-	-	-
9cen+/13stk+	-	-	-	-	1	1
inv(9)/15p+	1	-	1	-	-	-
inv(9)/inv17cen	-	1	1	-	-	-
13s+/13stk+	-	1	1	-	-	-
13stk+/14stk+	-	1	1	-	-	-
13stk+/16qh+	-	1	1	-	-	-
14stk+/15p+	-	1	1	-	-	-
14p+/22s+	1	-	1	-	-	-
14stk+/22stk+	1	-	1	-	-	-
15stk+/inv17cen	-	1	1	-	-	-
15stk+/22s+	-	1	1	-	-	-
15p+/ Yqh+	-	2	2	-	-	-
15stk+/Yqh-	-	1	1	-	-	-
16qh+/22stk+	2	-	2	-	-	-
16qh+/Yqh-	-	-	-	-	1	1
inv17cen/21stk+	-	1	1	-	-	-
1qh+/15p+/22p+	-	-	-	-	1	1
inv(9)/inv17cen/Yqh-	-	-	-	-	1	1
14stk+/15stk+/16qh+	1	-	1	-	-	-
total	**7 *(2.3%)***	**12 *(4.0%)***	**19 *(6.3%)***	**0**	**4 *(2.6%)***	**4 *(2.6%)***

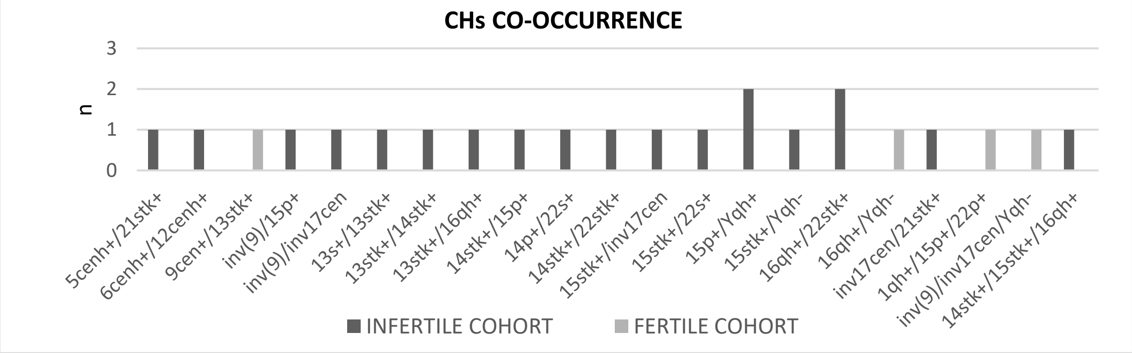

Supplementary Figure S1. Co-occurring pairs and trios of CHs in both cohorts. n = absolute number. A total of 19 (6.3%) co-occurrences were reported in the infertile cohort: 7 Females (2.3%) and 12 Males (4.0%); a total of 4 (2.6%) co-occurences were reported in the fertile cohort: all in 4 males (2.6%).

Supplementary Table S3. Distribution of different CHs in the infertile cohort according to clinical indications

**Clinical indications**	**1qh+**	**9 var**	**16qh+**	**Y var**	**13 var**	**14 var**	**15 var**	**21 var**	**22 var**	**Rare CHs**	**Total (n)** **CHs** *Carriers*
Primary infertility n=134	2	9	1	4	0	2	7	1	3	6	**35** *26 [19.4%]*
Secondary infertility n=20	2	1	2	0	0	2	1	0	2	0	**10** *8*
Recurrent pregnancy loss n=120	6	7	7	1	5	6	5	1	2	1	**41** *32 [26.7%]*
Medically assisted reproduction failure (≥1 cycle) n=53	3	0	1	3	0	1	2	0	1	0	**11** *9 [17.0%]*
Oligoasthenoteratozoospermia n=20	1	3	1	0	0	0	1	0	0	1	**7** *6*
Azoospermia n=11	0	0	0	1	0	0	0	0	1	0	**2** *2*
Premature ovarian failure n=9	0	0	1	n/a	0	1	0	0	1	0	**3** *2*
Primary amenorrhea n=1	0	0	0	n/a	0	0	0	0	0	0	**0** *0*
Secondary amenorrhea n=2	0	1	0	n/a	0	0	0	0	0	0	**1** *1*
Poor oocyte quality / Low ovarian reserve n=3	1	0	0	n/a	0	0	0	0	0	0	**1** *1*

The total number of heteromorphisms across clinical indications exceeds the total number of heteromorphisms detected in the study group due to the possible presence of multiple clinical indications in the same individual; n/a - not applicable; var – variants; grey lines indicate the top three clinical indications with the highest representation based on Pareto curve analysis; grey columns indicate the most frequent variants detected in the infertile cohort.
